# Femtosecond Laser Induced Lattice Deformation in KTN Crystal

**DOI:** 10.3390/mi13122120

**Published:** 2022-11-30

**Authors:** Quanxin Yang, Bin Zhang, Yuanbo Li, Xuping Wang, Feng Chen, Pengfei Wu, Hongliang Liu

**Affiliations:** 1 Institute of Modern Optics, Nankai University, Tianjin 300350, China; 2 School of Physics, State Key Laboratory of Crystal Materials, Key Laboratory of Particle Physics and Particle Irradiation, Shandong University, Jinan 250100, China; 3 Advanced Materials Institute, Qilu University of Technology (Shandong Academy of Sciences), Jinan 250014, China; 4 Tianjin Key Laboratory of Optoelectronic Sensor and Sensing Network Technology, Nankai University, Tianjin 300350, China; 5 Tianjin Key Laboratory of Micro-Scale Optical Information Science and Technology, Nankai University, Tianjin 300350, China

**Keywords:** femtosecond laser processing, relaxor ferroelectric material, field-induced lattice modification

## Abstract

In recent years, many novel optical phenomena have been discovered based on perovskite materials, but the practical applications are limited because of the difficulties of device fabrication. Here, we propose a method to directly induce localized lattice modification inside the potassium tantalate niobate crystal by using the femtosecond laser. This selective modification at the processed regions and the surrounding areas is characterized by two-dimensional Raman spectrum mapping. The spectrum variations corresponding to specific lattice vibration modes demonstrate the lattice structure deformation. In this way, the lattice expansion at the femtosecond laser irradiated regions and the lattice compression at the surrounding areas are revealed. Furthermore, surface morphology measurement confirms this lattice expansion and suggests the extension of lattice structure along the space diagonal direction. Moreover, the existence of an amorphization core is revealed. These modifications on the sample lattice can induce localized changes in physicochemical properties; therefore, this method can realize the fabrication of both linear diffraction and nonlinear frequency conversion devices by utilizing the novel optical responses of perovskite materials.

## 1. Introduction

In the past decades, the development of laser technology has promoted related research on laser–matter interaction, especially after the invention of the femtosecond laser [1,2,3]. With ultrashort pulse duration and ultrahigh peak power, the femtosecond laser can be utilized as an efficient tool in the modification of solid materials, such as polymer [4], dielectric [5,6,7,8,9], glass [10,11,12], ceramic [13], and even metal and alloy [14,15]. For the dielectric situation, the focused optical field can cause intense ionization inside the sample, and the energy transfer between the formed electronic plasma and the lattice system leads to the modification of the lattice structure [5]. Owing to the ultrashort property of the femtosecond laser, the thermal conduction to the surrounding regions is limited [16]. By employing the motorized stage or the galvo scanning system, the modified regions can be fabricated according to various three-dimensional structures.

Potassium tantalate niobate (KTa_1−x_Nb_x_O_3_, KTN) crystal has attracted much attention due to its marvelous properties such as high dielectric permittivity [17], large electro-optic coefficients [18,19], and superior optical nonlinear response [20,21,22,23,24,25]. As a compositional-disordered relaxor ferroelectric, KTN crystal not only possesses the controllable phase transition temperature but also shows novel relaxation phenomena during the phase transition [23]. Compared to the classical relaxor ferroelectrics, KTN, as a novel lead-free perovskite material, presents a relatively simple and clear lattice structure [26,27]. Thus, it is suitable for being the substrate for further investigations of lattice modification, relaxor ferroelectric, and its optical response.

In this paper, we report on the femtosecond laser induced lattice deformation in the KTN crystal. This selective modification of the irradiated regions and the surrounding areas is characterized by the Raman spectrum. The spectrum variations compared to the unprocessed bulk material demonstrate the elongation and expansion of the lattice structure. Further surface morphology measurement confirms this phenomenon and reveals the extension of lattice deformation along the space diagonal directions. In addition, the existence of an amorphization core is suggested. Such modification on the sample lattice can induce localized changes in physicochemical properties. With different structures designed, this method can realize the fabrication of different optical functional devices, especially frequency conversion devices with periodic structures, which are capable of integrated photonic circuits.

## 2. Methods

### 2.1. Sample Preparation

The KTN single crystal sample (with the Curie temperature T_C_ = 10 °C and the Nb concentration x = 0.357) used here is grown by the top-seeded solution growth (TSSG) method [28]. The raw sample is then cut into dimensions of 3.6 mm (x) × 3.0 mm (y) × 1.2 mm (z) with all the x × y and the y × z facets optically polished. The [100], [010], [001] crystallographic directions of this sample are along the x, y, and z axes, respectively.

### 2.2. Femtosecond Laser Modification

**Figure 1:**
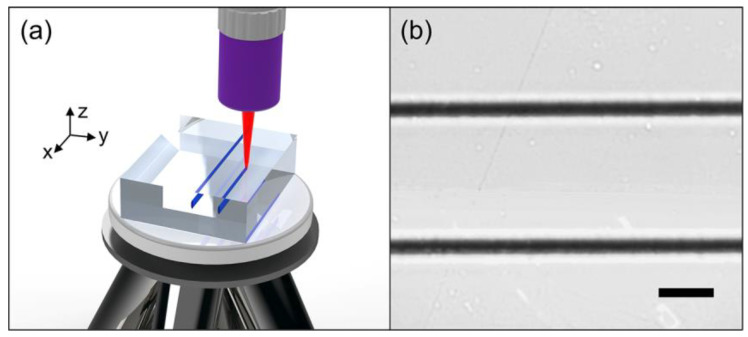
(**a**) The schematic diagram of femtosecond laser processing. Coordinate axes are defined here. (**b**) The microscope image of the processed KTN sample (x × y facet). The scale bar in (**b**) is 20 μm.

As for the femtosecond laser modification process, we utilize a fiber-coupled femtosecond laser system (FemtoYL-25, YSL Photonics Co., Wuhan, China) at Shandong University as the laser source. It generates linearly polarized 400 fs pulses with a central wavelength of 1031 nm and a repetition rate of 25 kHz. The single pulse energy and the polarization of the output laser are controlled by a Glan–Taylor Prism combined with a half-wave plate. During the whole experiment process, the polarization direction of the femtosecond laser is kept parallel to the scanning direction of the sample. The laser beam is focused by a microscope objective (50×, N.A. = 0.45) into the sample beneath one x × y facet. The maximum focal depth is fixed at ~30 μm beneath the selected surface. The sample is placed on a computer-controlled precise motorized stage and the scanning speed is set to 1 mm/s. The schematic diagram of femtosecond laser processing is shown in Figure 1a.

### 2.3. Confocal μ-Raman Measurement and Surface Morphology Analysis

The lattice structure differences between the modified regions and the non-processed bulk areas are characterized by a confocal μ-Raman spectroscopy system (XperRam200, Nanobase, Seoul, The Republic of Korea). The 532-nm laser beam with the power of 20 mW and the polarization along the x-direction is focused onto the surface (x × y facet) by a microscope objective (40×, N.A. = 0.75). The spectrometer with a spectral resolution of 0.8 cm^−1^ analyzes the collected back-scattered Raman signals. The confocal design provides higher vertical and horizontal resolutions; therefore, the galvanometric scanning system can realize the two-dimensional mapping of the Raman spectrum under a spatial resolution of 100 nm. The integral time of each measurement is set to 300 ms to guarantee spectral accuracy.

A three-dimensional optical profiler (NewView9000, Zygo Co., Middlefield, CT, USA) is utilized to analyze the surface morphology of the processed region under a sub-nanometer precision. To guarantee the accuracy of the results, signal over-sampling is enabled, and each measurement is triply averaged. Both the Raman measurement and the morphology analysis are performed at room temperature (~25 °C).

## 3. Results and Discussion

### 3.1. Microstructure

Under the experimental condition described in the Section 2 several modified tracks can be formed inside the sample. Figure 1b shows two typical tracks fabricated under the single pulse energies of 0.65 μJ and 0.71 μJ, respectively. These tracks present the appearance of typical type II modification in the field of femtosecond laser inscription. So-called type II modification refers to negative refractive index change (Δn < 0) caused by the localized lattice deformation with partial amorphization occurs [5]. In the meantime, the expanded lattice exerts stress on the surrounding crystalline substrates and causes densification in these areas. Detailed lattice dynamics are introduced later. Observing from the end-face through the microscope (Axio Scope A1, Carl Zeiss, Oberkochen, Germany), the vertical and horizontal widths of these modified tracks are around 25 μm and 9 μm, respectively. As a result, the track top is adjacent to the sample surface. Unlike the commonly used lithium niobate crystal [29], the KTN sample possesses a stronger threshold effect: when the single pulse energy is under a specific value (0.65 μJ in this case), the femtosecond laser will not cause any impact, while when the single pulse energy reaches this specific value, there will remain a distinct type II trace at the irradiated region. Even if the pulse energy is carefully adjusted around the threshold value, there is still no trace of type I modification.

### 3.2. Lattice Dynamics

For a perovskite crystal, the cubic lattice system with the *Pm3m* symmetry and the tetragonal lattice system with the *P4mm* symmetry are common [30]. The typical Raman spectrum of the KTN crystal conforms to the general regulation of the perovskite materials and consists of a dozen or more phonon modes, including the first-order and the second-order modes [31]. We mainly focus on these particular modes: the 2TA mode at 120 cm^−1^, which is caused by the TA-phonon-coupling at the Brillouin zone boundary and is sensitive to the lattice quality; the A_1_ + E(TO_2_) modes at around 201 cm^−1^, which is also the Fano resonance (one of the asymmetric line shapes) peak and associated with the vibrations of the Nb^5+^/Ta^5+^ ions and the spatial center of the O_6_^12−^ frameworks inside the NbO_6_^7−^/TaO_6_^7−^ octahedral units; the B_1_ + E(TO_3_) modes at 279 cm^−1^ and the A_1_ + E(TO_4_) modes at 554 cm^−1^, which represent the deformation of the O_6_^12−^ frameworks itself; the second-order mode at 578 cm^−1^, which is caused by the coupling of one TO_4_ phonon and one TA phonon.

With these classifications of phonon modes, the Raman spectrum of KTN crystal can be fitted by the combination of a Lorentzian central peak (CP), a Fano function, and the damped harmonic oscillator models as [32]:

(1) $$I(\omega )=\frac{{2A}_{\mathrm{CP}}}{\pi }\frac{{Г}_{\mathrm{CP}}}{{4\omega }^{2}{+Г}_{\mathrm{CP}}^{2}}{+I}_{B}(\omega )+\frac{I_{0}{\left(q+\varepsilon \right)}^{2}}{\left({1+\varepsilon }^{2}\right)}+\sum_{i}\frac{A_{i}{Г}_{i}\omega_{i}^{2}}{{\left(\omega^{2}-{\;\omega }_{i}^{2}\right)}^{2}{+\omega }^{2}{Г}_{i}^{2}},$$

where *A_CP_* and *Г_CP_* represent the CP intensity and its full width at half maximum (FWHM). *A_i_*, *ω_i_*, and *Г_i_* are the intensity, the frequency, and the damping constant of the *i*th Raman mode, respectively. *q* is defined as the asymmetry parameter. *ε*, the reduced energy, can be given by:

(2) $$\varepsilon =\frac{2(\omega \;-{\;\omega }_{{\mathrm{TO}}_{2}})}{{Г}_{{\mathrm{TO}}_{2}}}.$$

*I_B_* can be expressed as:

(3) $$I_{B}=P{\left(\omega \;-{\;\omega }_{{\mathrm{TO}}_{2}}\right)}^{3}+Q{\left(\omega \;-{\;\omega }_{{\mathrm{TO}}_{2}}\right)}^{2}+R{\left(\omega \;-{\;\omega }_{{\mathrm{TO}}_{2}}\right)}^{1}+S,$$

where *P*, *Q*, *R*, and *S* are all constants. *I_0_*, *ω_TO2_*, and *Г_TO2_* are the intensity, the frequency, and the FWHM of the Fano resonance peak, respectively.

**Figure 2:**
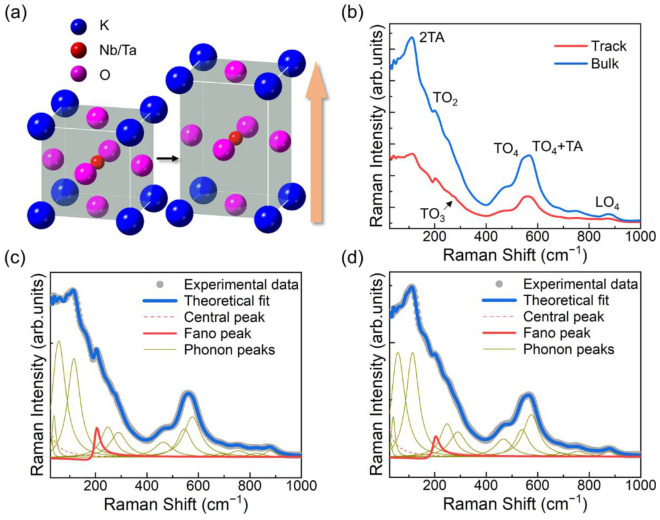
(**a**) The sketch diagram of the lattice deformation during the femtosecond laser processing. (**b**) The Raman scattering spectra of the femtosecond laser induced track (red line, and the bulk material (blue line), respectively. (**c**) Corresponding fitting curves of the track, and (**d**) the bulk material, respectively.

The modifications in lattice structure can cause variations in vibration modes, which results in the changes of Raman phonon modes in the spectrum. Based on the aforesaid analysis, the Raman spectra of the femtosecond laser processed region and the bulk material are fitted, as shown in Figure 2b–d. The relative intensity and position variations of the phonon modes before and after the femtosecond laser processing are distinctly exhibited. These variations can be concluded as follows: the intensity decrement and frequency softening of the 2TA mode; the resonance depth increment of the Fano resonance; the appearance of the TO_3_ modes; the intensity increase in the TO_4_ modes compared to the relatively stable TO_4_ + TA mode. In addition, the general intensity of the processed region also decreases.

**Figure 3:**
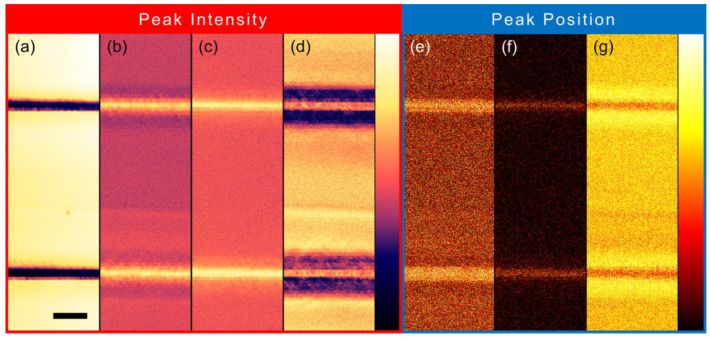
Two-dimensional spatial distributions of the Raman spectra representing (**a**–**d**) peak intensities and (**e**–**g**) peak positions obtained at room temperature. Channel details can be found in Table 1. The scale bar is 10 μm.

To further analyze the spatial differences of the lattice dynamics, a two-dimensional mapping of the Raman spectrum is performed. The imaging channels are carefully selected to maximize the contrast, and finally, seven channels (four for the intensity and three for the peak position) are defined in total, as shown in Table 1. The first channel represents the general intensity of the whole spectrum calculated by the integration method. The other three intensity-type channels are all defined as the ratios to eliminate the influence of absolute intensity change exhibited by the first channel. The second channel represents the relative Fano resonance depth and is defined as the intensity ratio of the TO_2_ modes peak at 201 cm^−1^ and the valley at 191 cm^−1^. At the same time, the third channel is defined as the intensity ratio of the TO_3_ modes peak at 278 cm^−1^ and the neighboring spectrum at 268 cm^−1^. The fourth channel is defined as the intensity ratio of the TO_4_ modes peak at 543 cm^−1^ and the second-order TO_4_ + TA mode at 570 cm^−1^. The fifth and sixth channels represent the peak positions of the TO_2_ modes and the TO_3_ modes, respectively. In contrast, the seventh channel is defined as the position of the higher peak between the TO_4_ modes and the TO_4_ + TA mode. The corresponding results are illustrated in Figure 3, and these patterns can confirm the aforesaid analyses of the Raman spectrum variations. Moreover, several new phenomena emerge: the surrounding areas present opposite variation tendencies for both the intensity and the peak position compared to the femtosecond laser processed track regions, as exhibited in Figure 3b,d,g; the peak redshifts (i.e., wavenumber increase) of the TO_2_ modes and TO_3_ modes, as shown in Figure 3e,f.

According to the vibrational modes behind the aforesaid Raman phonon modes, the variations on the spectrum correspond to specific lattice deformations. In such a way, the lattice dynamics during the femtosecond laser irradiation can be inferred as follows: the general intensity quench on the whole spectrum indicates the occurrence of lattice distortion; the intensity decrement and the frequency softening of the 2TA mode also suggest this distortion; the relative intensity enhancements of the Fano resonance, the TO_3_ modes and the TO_4_ modes demonstrate the intensification of the vibrations between the Nb^5+^/Ta^5+^ ions and the spatial center of the O_6_^12−^ frameworks, and the deformation of the O_6_^12−^ frameworks; these intensity increments of the relative motion inside the crystal lattice and the redshifts of the TO_2_ modes both indicate the lattice elongation along one specific direction (generally treated as the crystallographic direction [001]), as illustrated in Figure 2a. Longer bond length corresponds to a lower refractive index, which is the typical characteristic of type II modification. The lattice expansion that occurs at the femtosecond laser processed region results in lattice compression in the surrounding areas. Thus, opposite variation tendencies on the Raman spectrum appear.

### 3.3. Volumetric Expansion

**Figure 4:**
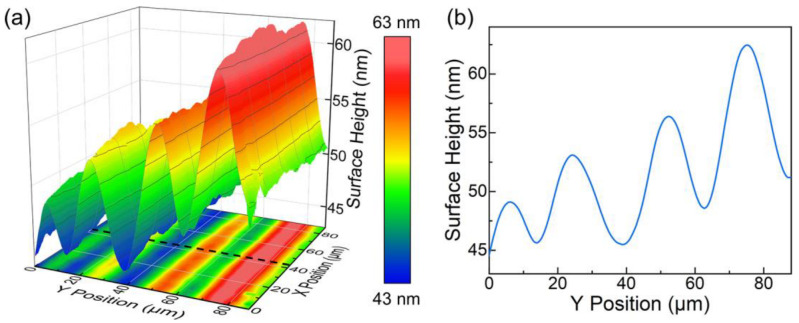
(**a**) Three-dimensional contour map of the femtosecond laser processed region on the x × y facet. The raw data are releveled according to other unprocessed regions of the sample surface. (**b**) The height profile along the black dotted line in (**a**).

The surface appearance at the femtosecond laser irradiated region is shown in Figure 4. The whole modified region, along with the lateral neighboring areas (~20 μm for both sides), has bulged for ~50 μm, but the noteworthy thing is that there exists subsidence right above the irradiated region compared to the neighboring areas, which is pretty counterintuitive.

The mechanism behind this surface undulation can be deconstructed into two parts: the ordinary uniform stress compression and the extension of the lattice deformation along one specific angle. As introduced above, when the specific region is irradiated by the femtosecond laser, the ionization-generated plasma can absorb energy from the pulse and further induce local amorphization. The volumetric expansion accompanying this process provides a stress field and compresses the surrounding material. As a result, this isotropic volumetric expansion spreads inside the sample and causes a uniform spherical deformation (a wide cylindrical deformation for the track situation) on the sample surface.

On the other hand, the crystalline material in the vicinity of the amorphization core suffers the largest squeezing stress; thus, a high density of defects and lattice imperfections are implanted during the femtosecond laser irradiation. As a result, lattice deformation occurs. It should be noticed that the three crystallographic directions are all equivalent for KTN crystal at the cubic phase. When the lattice deformation occurs, the elongation happens along one specific crystallographic direction randomly. As a result, this lattice deformation can extend along the space diagonal directions of the x, y, and z axes to minimize the Gibbs free energy. In this way, such localized lattice deformation induced by the femtosecond laser can spread and generate anisotropic volumetric expansion inside the sample. As a result, dotted deformations at the extended diagonals (two narrow strip-shaped deformations at both sides for the track situation) emerge on the sample surface. The final surface appearance is decided by these two factors. Thus, the track fabricated under higher energy presents more intense and extensive lattice deformation, resulting in the base-line-like increased tendency of the surface height exhibited in Figure 4b.

## 4. Conclusions

In this paper, a femtosecond laser induced lattice deformation in KTN crystal has been realized. A strong threshold effect of the KTN sample is revealed, and the damage threshold of KTN crystal is 0.65 μJ/pulse under our experimental condition. This selective modification of the processed regions and the surrounding areas is characterized by the two-dimensional Raman spectrum mapping. With the spectral fitting method, the spectrum variations compared to the unprocessed bulk material are clearly presented. These spectrum variations correspond to specific lattice structure deformation, i.e., the lattice elongation along one crystallographic direction. Furthermore, surface morphology measurement confirms this lattice expansion and reveals the extension of lattice deformation along the space diagonal direction. Moreover, the existence of an amorphization core is suggested. Such modification on the sample lattice can induce localized changes in physicochemical properties. With different structures designed, this method can realize the fabrication of various optical functional devices, especially frequency conversion devices with periodic structures, which are capable of integrated photonic circuits.

## Figures and Tables

**Table 1:** The channel definitions used for the two-dimensional imaging of Raman spectra.

Channel Number	Figure 3	Channel Type	Peak (cm^−1^)	Calculation Method	Minimum Value	Maximum Value
1	(a)	Intensity	Whole Spectrum	\	0.42	1 (Normalized)
2	(b)	Intensity	201 (TO_2_)	(201)/(191)	0.94	1.084
3	(c)	Intensity	278 (TO_3_)	(278)/(268)	0.85	0.993
4	(d)	Intensity	543 (TO_4_)	(543)/(570)	0.92	0.988
5	(e)	Shift	201 (TO_2_)	\	197.5 cm^−1^	205.8 cm^−1^
6	(f)	Shift	278 (TO_3_)	\	274.3 cm^−1^	281.6 cm^−1^
7	(g)	Shift	543&570 (TO_4_&TO_4_ + TA)	\	544.0 cm^−1^	579.0 cm^−1^

## Data Availability

The data presented in this study are available from the corresponding author upon reasonable request.
